# Effects of Blink Rate on Tear Film Optical Quality Dynamics with Different Soft Contact Lenses

**DOI:** 10.1155/2019/4921538

**Published:** 2019-07-09

**Authors:** María García-Montero, Laura Rico-del-Viejo, Irene Martínez-Alberquilla, Jose Luis Hernández-Verdejo, Amalia Lorente-Velázquez, David Madrid-Costa

**Affiliations:** Optometry and Vision Department, Faculty of Optics and Optometry, Complutense University of Madrid, Madrid, Spain

## Abstract

**Objective:**

The aim of this study was to investigate tear film optical quality dynamics for four types of silicone hydrogel contact lenses (SHCLs) for daily wear over a 15-day period and for different blink rate (BR) patterns.

**Methods:**

A prospective randomized, double-blind, cross-over pilot study including four SHCLs (A: lotrafilcon B (Air Optix plus HydraGlyde, Alcon Laboratories); B: samfilcon A (Ultra, Bausch & Lomb); C: comfilcon A (Biofinity, CooperVision); and D: filcom V3 (Blu:gen, Mark'Ennovy)). Serial measurements of Objective Scatter Index (OSI) using the HD Analyzer (Visiometrics S.L., Terrassa, Spain) were taken at different blinking patterns: blinking every 2.5 seconds (high BRs) and every 9 seconds (low BRs). They were performed during the first visit before CL insertion (baseline), after 20 minutes of CL wear (Day 1), and during the last visit after 8 hours of CL wear on day 15 of use (Day 15).

**Results:**

Normal young healthy subjects were recruited and fitted with the four lenses. For low BRs, the mean OSI value increased over time for all CLs and the slope of the curve also increased for all CLs, except for CL D. However, for high BRs, the mean OSI value increased only for CLs B and C and the slope of the curve did not change over time for any of them.

**Conclusions:**

These results suggest that the tear film optical quality dynamics after wearing SCHLs for 15 days seems to undergo a slight deterioration only for lowest BR.

## 1. Introduction

The tear film plays an important role in the optical quality of the human eye [1–4]. Several authors have studied changes in tear film quality over time in contact lens wearers [5–10], using dynamic-area high-speed videokeratoscopy [5, 6, 9, 10] and a double-pass method [7]. The results show that there is a significant decrease in prelens tear film quality with respect to the baseline precorneal tear film quality with monthly hydrogel and silicone hydrogel contact lenses (CLs) over one week of use [5]; daily CLs (delefilcon A silicone hydrogel and omafilcon A hydrogel) over 4 hours of use [6]; daily hydrogel CLs over one day of use [9]; hard CLs (PMMA), rigid gas permeable (RGP) CLs (Boston XO), and soft silicone hydrogel CLs over one day of use [8]; and HEMA multifocal CLs over one day of use [7].

New CL materials currently focus on improving tear film stability in order to provide optimal vision quality. At the same time, the use of desktop, laptop, and tablet computers, smartphones, and electronic reading devices has become ubiquitous with today's society [11]. Under these conditions, the blink rate (BR) decreases, creating a risk factor for ocular exposure [12]. Additionally, CL wearers tend to increase their BRs, presumably because of surface irritation from the lens or unstable tear film [13]. All these above-mentioned conditions can affect the optical quality of the tear film and consequently the success of CL adaptation.

The High Definition Analyzer (HD Analyzer™) (Visiometrics S.L., Terrassa, Spain) is an instrument that uses a double-pass method that was developed to perform an objective evaluation of optical quality. It determines the Objective Scatter Index (OSI) using the point spread function (PSF), which determines how a point source of light is imaged on the retina. The OSI is an objective index of intraocular scattered light. It is a parameter that has been used for assessment of the dynamics of the human tear film in dry eyes or normal eyes [14–17]. Thus, an indirect approach can be used to quantify tear film quality based on dynamic analysis of OSI values [18]. However, there are not studies that reported OSI dynamic changes in silicone hydrogel CL wearers. Applying the dynamic analysis of OSI values is a novel approach in the study of the behavior of different contact lenses with low and high blinking patterns.

The aim of the current study was to evaluate the tear film optical quality dynamics for four types of silicone hydrogel contact lenses (SHCLs) over 15 days of wear for different BR patterns.

## 2. Materials and Methods

### 2.1. Subjects

A total of 15 subjects (12 men and 3 women; mean age 24.1 ± 2.2 years; age range 29 to 21 years) took part in this study. The study was carried out at the Faculty of Optics and Optometry of the Complutense University of Madrid. It was reviewed and approved by the Institutional Review Board of the San Carlos University Hospital in Madrid. It was conducted in accordance with the Declaration of Helsinki. All the subjects gave informed consent and agreed to all the procedures after being informed in detail about the nature of the study. Inclusion criteria were age range of 20 to 30 years, current CL wearers, cylinder refractive error <0.50 D, and spherical refractive error range of +4.00 to −4.00 D. Exclusion criteria included active ocular allergy and refractive surgery or systemic medication known to affect tear film production.

### 2.2. Optical Quality Analysis System: High Definition Analyzer

Optical quality was evaluated using the HD Analyzer™ (Visiometrics S.L., Terrassa, Spain). This instrument, based on the double-pass method, provides an objective clinical evaluation of the eye's optical quality. It was designed for use in clinical practice to objectively determine the optical quality of the human eye, including intraocular scattering, using a double-pass method.

The OSI is a parameter that allows intraocular scattered light to be evaluated objectively. It is computed by evaluating the amount of light on the periphery of the double-pass image in relation to the amount of light at its centre. The central area selected was a circle with a radius of 1 minute of an arc, while the peripheral zone was a ring set between 12 and 20 minutes of an arc. As the OSI value increases, the level of intraocular scattering also increases [17].

The “Tear Film Analysis” program included in the commercially available software was used to record dynamic changes in OSI values. This program consists of a 20-second examination with an OSI measurement every 0.5 seconds that gives a quantitative and objective evaluation of the loss of optical quality due to tear film degradation. The HD Analyzer™ (Visiometrics S.L., Terrassa, Spain) system allows to monitor the dynamic changes in optical quality. The result screen shows all images recorded during the process, with one OSI value for each image. When the subject blinked, the OSI value was replaced by a blink mark and no value was recorded at this point.

### 2.3. Measurement of Dynamic Optical Quality

The subject's spherical refractive error was automatically corrected by the HD Analyzer™ (Visiometrics S.L., Terrassa, Spain).

The OSI dynamic measurements were taken in two different situations after a 5-minute period of dark adaptation. Two blink patterns had been defined based on how often the subject was allowed to blink. They were asked to blink every 2.5 seconds (high BRs) during the whole recording process (20 seconds), and finally, they were asked not to blink for 9 seconds (low BRs). Blink rates were controlled by an audible signal that the instrument emits. Before starting, the registration subjects were instructed to blink twice naturally and then keep their eyes open. There was a wash-out period of 10 minutes between both measurements (high and low BRs).

### 2.4. Study Protocol and CL Types

**Figure 1:**
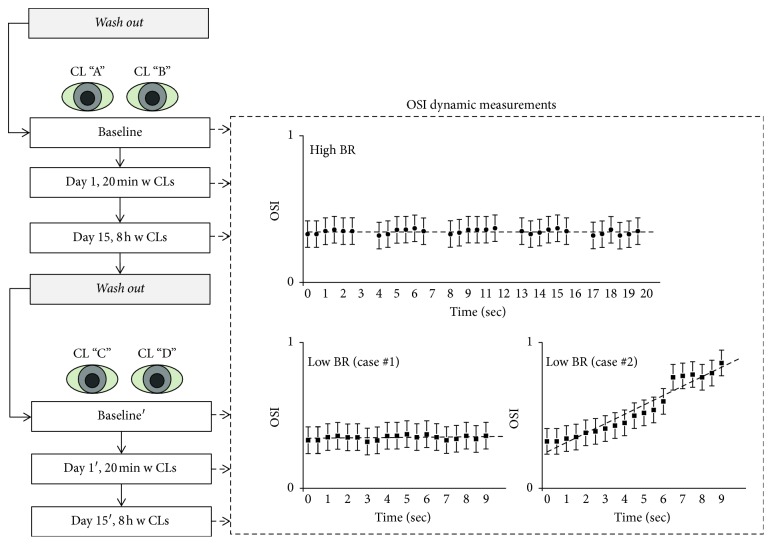
Clinical protocol. Repeated-measures ANOVA of the Objective Scatter Index (OSI) was performed for two different blinking patterns: blinking every 2.5 seconds (high BRs) and not blinking for 9 seconds (low BRs). The graphic representation of OSI values for high BRs was recorded over 20 seconds. Successive intervals were registered separated by blinks, and the graph represents a stability model of the OSI value with a flat curve. The graphic representation of OSI values for low BRs was recorded over 9 seconds, and only one interval was registered and analyzed. The distribution of OSI values over time has different behaviors: case #1 represents a stable model with a flat curve, while case #2 represents a model of instability with an increase in the slope of the curve. Measurements were performed for each visit (baseline, Day 1, and Day 15) using randomly assigned contact lenses. OSI: Objective Scatter Index; dyn.: dynamic; BR: blink rate; CL: contact lens; w CLs: wearing contact lenses; sc: seconds.

This is a prospective, randomized, double-blind, cross-over pilot study (see Figure 1 for a detailed explanation of the protocol). It was conducted over five consecutive weeks, using 4 types of monthly CLs made of silicone hydrogel (SH) material for daily wear. The lens parameters are shown in Table 1.

Previously, one week of wash out without any CL was given to participants. During the first two weeks, each subject used one CL in the right eye and another different CL in the left eye. After a week of wash out, another two CLs were assigned to the right and the left eye for two more weeks. CLs were assigned randomly, and subjects were instructed to wear the CLs for 8 hours a day. Slit lamp assessment was performed after one week of wash out without any CL and after 15 days of use of the two pairs of CLs assigned.

The OSI dynamic measurements (high and low BRs) were taken for each pair of CL on the first visit and last day of wear (15 days). On the first day of wear, measurements were taken before CL insertion (baseline) and at 20 minutes of wear (Day 1). On the last day of wear (after 15 days of use), measurements were taken at 8 hours of wear (Day 15). All subjects used the same solutions to care for the lenses (OptiFree Express MDS; Alcon Laboratories, Inc., Fort Worth, TX, USA). Subjects were exposed to the controlled environmental conditions, temperature 24 ± 2°C and humidity 38 ± 2%, before being examined for 10 minutes.

### 2.5. Statistical Analysis

Statistical analyses were performed using SAS software, version 9.4 (SAS Institute, Inc., Cary, NC, USA). The data exported from the HDA contained 40 OSI values with the “Tear Film Analysis” program. The software registered an OSI value every 0.5 seconds for 20 seconds. For high-BR situations, all values were analyzed, but for low-BR situations, only the first 9 seconds were analyzed. Statistical analysis was performed by descriptive analysis to obtain the mean results and standard deviations. In order to assess tear film stability, the relationship between OSI values and time was analyzed by regression models and the slope of the curve was calculated. Therefore, the mean and standard deviation were analyzed for the OSI dynamic values and slope of the curve for each BR situation registered. The Shapiro–Wilk test for normality was applied followed by the appropriate parametric ANOVA test with the Greenhouse–Geisser correction or nonparametric Friedman test to compare the effects of time (comparison between visits). The significance level used was *p* < 0.05.

## 3. Results

The baseline OSI values were 0.31 ± 0.16, 0.46 ± 0.13, 0.34 ± 0.08, and 0.40 ± 0.13 for CLs A, B, C, and D, respectively. There were not differences between them (*p*=0.10). No adverse events on the ocular surface occurred during the study.

### 3.1. OSI Dynamic Analysis for Low-BR Situations

**Figure 2:**
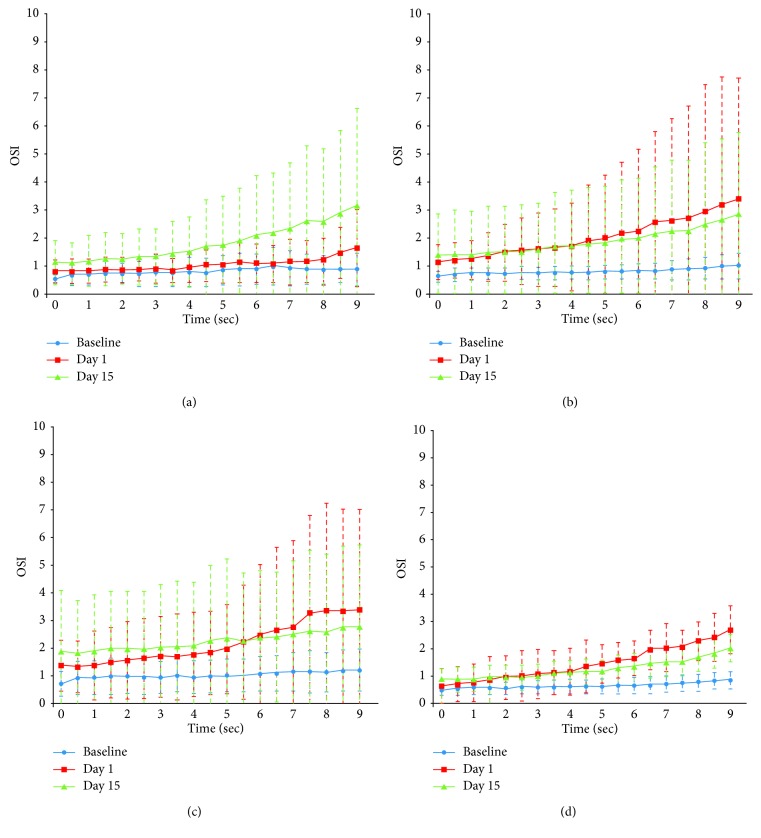
Graphic representation of OSI dynamics for low BRs. OSI: Objective Scatter Index; dyn.: dynamic; BR: blink rate; CL: contact lens. (a) CL A. (b) CL B. (c) CL C. (d) CL D.

A summary of the results is shown in Table 2. Repeated-measures ANOVA on the effect of the visits (baseline, Day 1, and Day 15) showed the effect of CL wear over time on OSI dynamics. The mean OSI dynamic value increased for all CLs. The baseline slope of the curve showed values between 0.03 ± 0.05 (CL A) and 0.04 ± 0.07 (CL C). The slope of the curve also increased for CLs A and B and for CLs C and D, and there was no statistical significance (Figure 2).

### 3.2. OSI Dynamic Analysis for High-BR Situations

**Figure 3:**
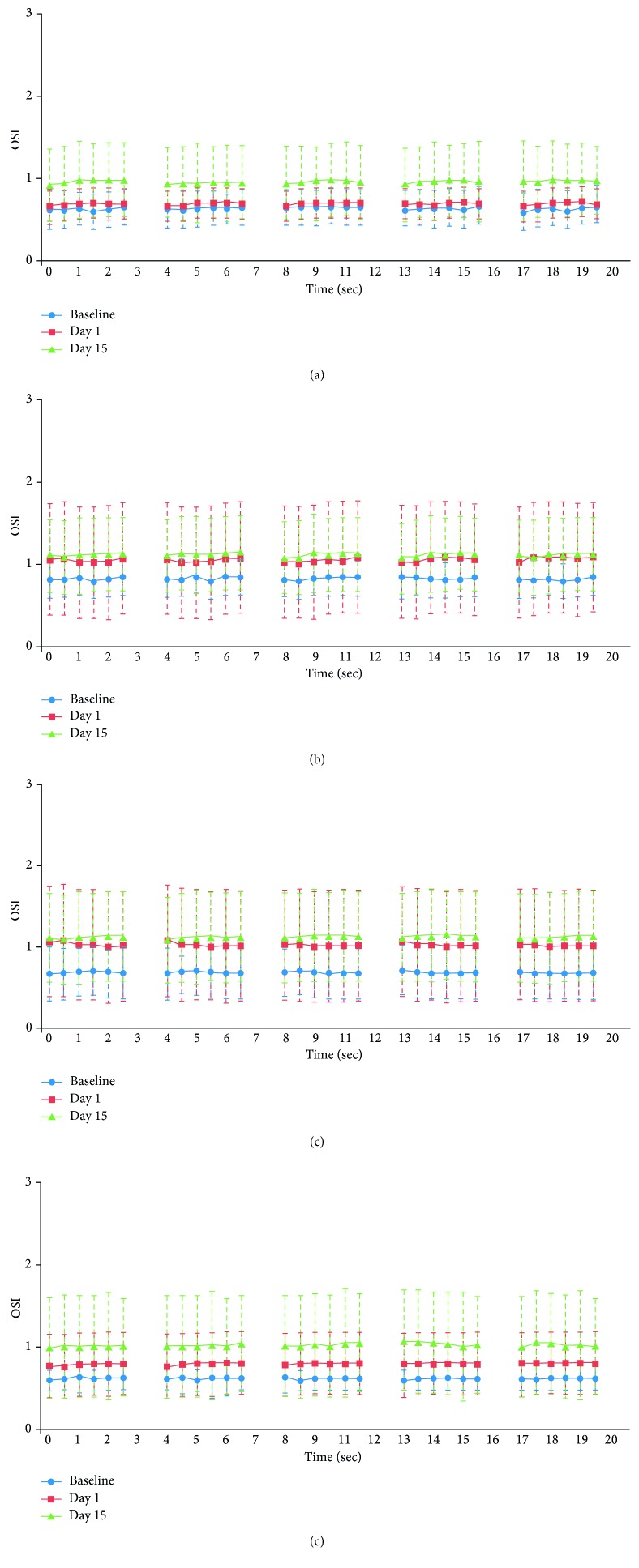
Graphic representation of OSI dynamics for high BRs. OSI: Objective Scatter Index; dyn.: dynamic; BR: blink rate; CL: contact lens. (a) CL A. (b) CL B. (c) CL C. (d) CL D.

Analysis statistics for OSI dynamics are summarized in Table 3. The OSI dynamic score increased (optical quality decreased) over time for all CLs, although the changes were statistically significant only in CLs B and C (*p* < 0.05). Nevertheless, the stability of the curve for different interval measurements during the 20 seconds recorded showed a flat slope for all CLs (Figure 3).

## 4. Discussion

A CL compartmentalizes the tear film, isolating the mucin layer behind the lens, thinning the prelens tear film, and disrupting the lipid layer [19–22]. Various studies have shown that CLs induce tear film changes and play an important role in the maintenance of optical quality [23]. This could potentially influence CL tolerance and lead to blurriness or fluctuations in vision. New designs for CLs focus on improving some of their properties, such as wettability [24] and lubricity, in order to provide the best quality vision and keep it stable over time, even under the more stressful conditions associated with the use of desktop, laptop, and tablet computers, smartphones, and electronic reading devices.

The aim of this cross-over pilot study was to assess the dynamic changes of optical quality during CL wear in a normal sample of participants. All of them wore 4 different types of SHCLs for 15 days.

The evidence suggests that the average OSI value in a healthy population is 0.7 ± 0.3 [17], 0.47 ± 0.11 [25], 0.41 ± 0.18 [15], and 0.32 ± 0.13 [26]. These results are in agreement with the average OSI value obtained in the current study. This observation reinforces the healthy characteristics of the eyes of the participants in the present study. Furthermore, it is important to assess the repeatability of the OSI. A sample classified by an ordinal scale depending on the increment of the OSI dynamic after each blink with the high-BR pattern revealed a quadratic kappa agreement *k* of 0.59 (95% CI 0.44 to 0.74) [27]. Regarding the OSI static value, the limit of repeatability was 0.26 (56.1%) [25] and 0.11 (34.4%) [26]. The tear film is generally evaluated in terms of quality and quantity, which are essential factors when characterizing tear film dynamics [28]. Regardless of the tear film quality, the tear film stability parameters in the present study were defined based on the OSI dynamic value and the slope of the curve. The ability of the prelens tear film to retain a smooth refractive surface on the CL surface was altered for some different types of CLs and even more so for low BRs. In fact, the variability of the OSI measurement increased for materials B and C, although it was more dramatic for low-BR patterns compared to high-BR patterns in the current pilot study.

A previous study shows that the best optical quality studied objectively by measuring the modulation transfer function (MTF) of the anterior surface of the film in the baseline situation was reached between 6 and 7 seconds after blinking, after which there was a progressive decrease [29]. However, the current study did not show a decrease in baseline optical quality. All participants had stable optical quality in the baseline situation under both BRs studied; OSI dynamic values were <1.00, and the slope of the curves was flat (the maximum value was 0.04 ± 0.07).

This situation changed when a CL was fitted. Both the types of CLs and the blink rate affect these results, which have been confirmed by other authors [5–10]. These results are also similar for non-contact lens wearers who suffer from dry eye disease (DED) [14].

In addition, it has been reported that CL wearers tend to increase their BRs, presumably because of surface irritation from the lens or unstable tear film [13]. Therefore, in order to determine optical quality during CL wear, it is interesting to analyze it for different BRs.

It is widely known that one of the functions of blinking is to reestablish a stable tear film, so it seems reasonable to assume that thinning tear film may induce poor quality of vision. A high blink rate has been evidenced in conversation situations (21.5 ± 5.6 blinks per minute), while a low blink rate in attention tasks and digital device situations [30].

Furthermore, spontaneous eye blinking has been found to be significantly reduced during activities such as reading, computer work, or other visual tasks requiring concentration [31, 32]. Many types of computers are currently used in everyday life. In most parts of the world, it is impossible to use a product or service that does not involve the use of a computer. In this way, the current results obtained by reducing the blink rate in CL users showed a worsening behavior in the stability of tear film optical quality compared to those obtained with high BRs. Table 2 shows that, for low BRs, there was an increase in the OSI dynamic value on day 15 of use for all CLs with respect to the baseline precorneal condition, except for CL D. There was a significant decrease in prelens tear film optical quality dynamics that was reflected in the slope of the curve.

However, for high-BR situations, the slope of the curve for all CLs remained constant. Despite the fact that, for higher BRs, the stability of the OSI value was maintained, there were observed changes in the OSI value. That is, after 15 days of use of CLs B and C, the OSI value worsened with respect to the baseline condition but stability was maintained. It is more than likely, however, that this finding has no negative impact on the participants' visual quality. The mean OSI values on Day 15 for high BRs were 0.93 to 1.15, in the limit of normal values of OSI. These findings suggest that all CLs studied provided optimal optical quality stability for high-BR situations over 15 days of use.

A CL is defined as a nonpathological factor impacting the tear film [33], and it is interesting to compare the effect of different blink rates over time. We have observed that, with a high BR, CL wear time does not cause a decrease in optical stability. In contrast, in conditions where the BR decreases, optical stability decreases as CL wear time increases.

Primarily, the CLs which provide worse optical quality stability could provide visual disturbance (fluctuation of vision). Depending on the lens, this could be more important after 15 days of using and/or for low BRs. Secondarily, visual disturbance could affect the subjective outcomes [34]. The results of the current study have 71% power to detect a difference in mean OSI values of 0.350, assuming a standard deviation in difference of 0.500, using a paired *t*-test with a 0.050 two-sided significance level. So, this result should be taken with caution even more considering that it is a pilot study.

It is difficult to assess the stability of tear film optical quality during CL wear with standard clinical examinations. With the results of the present study, the aim is to work on CL materials to provide better optical quality over the lifetime of the CL or reconsider CL replacement times in order to assess tear film stability and efficiency over time. Contact lens manufacturers improve wettability by increasing the surface energy and polarity of the lenses by coating, surface pretreatment, or incorporation of hydrophilic groups [24]. These conditions can improve the tear film surface quality and in consequence report a higher optical quality.

Dynamic models of optical changes in the human eye based on OSI values explain changes in vision due to tear film changes in normal eyes [14] and dry eyes [16, 35, 36]. It would be relevant to study this in the presbyopic population, which has poorer tear film stability when compared to the younger population [37] and needs more complex multifocal CL designs.

## 5. Conclusions

The parameters developed in this study show the difference in the dynamic behavior of OSI values between baseline and SHCL wear conditions and the influence of the BR on the shape of the curve. The final results show that CLs disrupt the tear film and increase OSI scores. Research is now focused on the properties of CLs, such as wettability, dehydration, lubricity, and modulus, and antimicrobial surface treatment, with the aim of providing the best vision quality, defined as optical quality stability between blinks over time.

## Figures and Tables

**Table 1:** Marketed silicone hydrogel contact lenses used in the study.

	CL A	CL B	CL C	CL D
Material	Lotrafilcon B	Samfilcon A	Comfilcon A	Filcom V3
Laboratory	Alcon	Bausch & Lomb	CooperVision	Mark'Ennovy
Commercial name	Air Optix plus HydraGlyde	Ultra	Biofinity	Blu:gen
Base curve (mm)	8.60	8.50	8.60	6.50–9.80 (step 0.30)
Diameter (mm)	14.20	14.20	14.00	11.50–16.50 (step 0.50)
Oxygen transmissibility (Dk/t)	138	163	160	50
Water content (%)	33	46	48	75
Modulus (MPa)	1.0	0.70	0.75	0.25

**Table 2:** Analysis statistics for dynamic Objective Scatter Index (dyn. OSI) for low blink rates.

		Low blink rate (mean ± SD)			
		Baseline	Day 1	Day 15	*p* value
Dyn. OSI	CL A	0.83 ± 0.46	1.07 ± 0.61	1.84 ± 1.64	0.04^*∗*^
	CL B	0.79 ± 0.26	2.02 ± 2.28	1.88 ± 1.98	0.02^*∗*^, 0.03^†^
	CL C	1.02 ± 0.60	2.16 ± 2.04	2.23 ± 2.44	0.05^*∗*†^
	CL D	0.66 ± 0.16	1.24 ± 0.63	1.10 ± 0.43	0.03^†^

Slope	CL A	0.03 ± 0.05	0.07 ± 0.08	0.22 ± 0.32	0.05^*∗*^
	CL B	0.03 ± 0.04	0.26 ± 0.58	0.15 ± 0.36	0.05^*∗*†^
	CL C	0.04 ± 0.07	0.25 ± 0.60	0.11 ± 0.13	0.06
	CL D	0.03 ± 0.05	0.09 ± 0.17	0.07 ± 0.10	0.629

^*∗*^Significant difference noted between baseline and Day 15; ^†^significant difference noted between baseline and Day 1; Day 1: after 20 minutes of CL wear; Day 15: after 8 hours of CL wear on day 15 of use; significant *p* value <0.05.

**Table 3:** Analysis statistics for dynamic Objective Scatter Index (dyn. OSI) for high blink rates.

	Dyn. OSI (mean ± SD)			
	Baseline	Day 1	Day 15	*p* value
CL A	0.66 ± 0.26	0.70 ± 0.20	0.93 ± 0.61	0.06
CL B	0.79 ± 0.22	1.12 ± 0.79	1.15 ± 0.58	0.02^*∗*^
CL C	0.70 ± 0.34	1.07 ± 0.77	1.15 ± 0.76	0.01^*∗*^
CL D	0.64 ± 0.16	0.82 ± 0.45	1.01 ± 0.67	0.08

^*∗*^Significant difference noted between baseline and Day 1; Day 1: after 20 minutes of CL wear; Day 15: after 8 hours of CL wear on day 15 of use; significant *p* value <0.05.

## Data Availability

The data that support the findings of this study are not publicly available because they contain information that could compromise research participants' privacy/consent.
